# Phylogenetic Relationships and Evolutionary History of Goats (Mammalia: *Capra*) From Türkiye and Iraq, Inferred From Complete Mitochondrial Genomes

**DOI:** 10.1002/ece3.71985

**Published:** 2025-08-13

**Authors:** Saffet Teber, Husham Abdulrahman Mahdi Al‐Abbasi, Perinçek Seçkinozan Şeker, Klaus‐Peter Koepfli, Ahmet Yesari Selçuk, Mehmet Baran, Coşkun Tez, Osman Ibiş

**Affiliations:** ^1^ Department of Agricultural Biotechnology, Faculty of Agriculture Erciyes University Kayseri Türkiye; ^2^ Genome and Stem Cell Center, GENKOK Erciyes University Kayseri Türkiye; ^3^ Department of Agricultural Science and Technologies, Graduate School of Natural and Applied Sciences Erciyes University Kayseri Türkiye; ^4^ Department of Forestry, Artvin Vocational School Artvin Çoruh University Artvin Türkiye; ^5^ Smithsonian‐Mason School of Conservation George Mason University Front Royal Virginia USA; ^6^ Department of Agricultural Biotechnology, Graduate School of Natural and Applied Sciences Erciyes University Kayseri Türkiye; ^7^ Department of Biology, Faculty of Sciences Erciyes University Kayseri Türkiye; ^8^ Vectors and Vector‐Born Diseases Research and Implementation Center Erciyes University Kayseri Türkiye

**Keywords:** *Capra*, domestication, Fertile Crescent, mitogenome, phylogeography, *rupicapra*

## Abstract

This study investigated genetic diversity, phylogenetic relationships, and evolutionary history of domestic goats from Türkiye and Iraq, along with wild goat and chamois species, using newly obtained mitogenomic sequences. Phylogenetic and phylogeographic analyses revealed a complex genetic structure among domestic goats, shaped by widespread distribution and gene flow. While haplotype A was predominant among domesticated breeds from both Türkiye and Iraq, haplotype G was also detected in the Turkish breeds. Notably, Turkish samples exhibited relatively higher nucleotide diversity (0.00133) compared to those from Iraq (0.00081), indicating greater genetic variability in the former population. Wild goat populations in Türkiye were clustered into two distinct lineages: (i) the Aegagrus lineage included the Artvin sample, some ancient genomes from the Taurus Mountains, and Iranian goats, and (ii) the Caucasian lineage contained Konya and Antalya samples, and some ancient genomes from the Taurus Mountains that were clustered closely with wild goats from the Caucasus. These findings suggest that geographic and ecological factors, such as the Anatolian Diagonal, influenced their diversification. Divergence time analyses indicated that the Caprinae began diversifying approximately 8.18 Mya, with initial splits in the *Capra* occurring around 3.22 Mya during the climatic fluctuations of the Late Pliocene/Early Pleistocene. The study also estimated the divergence of *C. aegagrus* and *C. hircus* at approximately 0.89 Mya in the Calabrian, with genetic diversification within domestic goats commencing 0.29 Mya in the Chibanian. The results provided robust evidence supporting Türkiye's role as a significant genetic center for goat domestication during the Neolithic period (~10,000 years ago). This hypothesis was further supported by the widespread presence of the common haplotype A in domestic goats, the high genetic diversity observed among domestic goats, and the region's proximity to the Fertile Crescent. The study underscored the importance of comprehensive genetic analyses in elucidating the evolutionary processes underlying goat domestication and highlighted the necessity for larger datasets and additional molecular markers to resolve the taxonomic complexities of wild goat populations in Türkiye, Iraq, and surrounding regions.

## Graphical abstract

This study explores the genetic diversity, phylogenetic relationships, and evolutionary history of domestic ( *Capra hircus* ) and wild goats ( *Capra aegagrus* ), as well as chamois ( *Rupicapra rupicapra* ) from Türkiye and Iraq, using complete mitochondrial genomes. The findings reveal two distinct wild goat lineages in Türkiye, shaped by geographic factors such as the Anatolian Diagonal, and highlight Türkiye's role as a key genetic center for goat domestication during the Neolithic period. Divergence time estimates suggest that *C. aegagrus* and *C. hircus* split around 0.89 million years ago, reinforcing the evolutionary significance of the region.

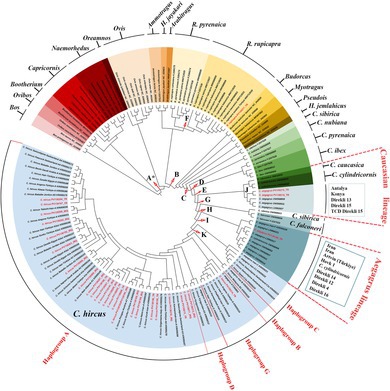

## 1 Introduction

The genus *Capra* belongs to the subfamily Caprinae within the family Bovidae (Artiodactyla: Mammalia) and it comprises domestic goats as well as their wild relatives, including species such as bezoars, turs, markhors, and ibex. These animals are found across a broad geographic range that spans from Siberia, North Africa, and the Middle East to the Caucasus, Anatolia, the Alps, and Iberia. Their ability to live in harsh environments such as rocky, mountainous regions and deserts allows them to survive even under pressure from predators (Geist 1987). Systematic studies on *Capra* species have been primarily based on traits such as horn morphology, body size, and variation in either nuclear or organelle DNA sequences (Manceau et al. 1999; Pidancier et al. 2006; Kazanskaya et al. 2007). However, these studies have led to differing conclusions, particularly regarding the evolutionary relationships within the genus, and, as such, there is still controversy in their classification (Manceau et al. 1999; Pidancier et al. 2006; Kazanskaya et al. 2007). Despite the ongoing debates, a widely accepted classification today includes nine wild goat species ( *C. aegagrus* , *C. sibirica* , *C. falconeri* , *C. caucasica* , *C. cylindricornis* , *C. ibex* , *C. pyrenaica* , *C. nubiana* , and *C. walie* ), and the domestic goat ( *C. hircus* ) (Pidancier et al. 2006).

*C. hircus* is known as one of the first livestock species to be domesticated (Zeder 2008). The domestication of the goat is considered one of the first milestones in human history because of contributing significantly to the agricultural revolution of the Neolithic period (Colli et al. 2015). Therefore, molecular studies on the genus *Capra*, using large sample sizes, have primarily focused on illuminating the processes of goat domestication and then determining their systematics, phylogeography, and the time of evolutionary divergence relative to wild goat species (Luikart et al. 2001; Naderi et al. 2007, 2008; Colli et al. 2015). The domestication of *C. hircus* dates back to approximately 10,000 to 11,000 years ago (Clutton‐Brock 1999; Zeder and Hesse 2000; Peters et al. 2005; Zheng et al. 2020). In this context, archaeological findings have shown that the wild ancestor of the domestic goat is the bezoar ( *C. aegagrus* ), native to the mountainous regions of the Middle East, particularly the Fertile Crescent, an area that extends from Southeastern Anatolia (Türkiye) to the Zagros Mountains in Central Iran. Although the existence of evidence of mitochondrial introgression or the claim that ongoing interspecies gene flow has occasionally complicated this issue, this finding is robustly supported by molecular evidence based on nuclear and mitochondrial gene sequences (Takada et al. 1997; Manceau et al. 1999; Zeder and Hesse 2000; Luikart et al. 2001; Vigne et al. 2005; Zeder et al. 2005; Pidancier et al. 2006; Naderi et al. 2007, 2008; Colli et al. 2015).

With the global spread of humans throughout the domestication process and beyond, over a thousand domestic goat breeds are known today, each exhibiting distinct or mixed genetic structures and diverse demographic histories (FAO 2015). The total genetic variation between domestic goats across different continents is quite low, and only 10% of the mitochondrial DNA (mtDNA) variation in goats is partitioned between continents (Luikart et al. 2001). Despite this mixed genetic structure leading to a weak phylogenetic and phylogeographic signal for evolutionary studies (Colli et al. 2018; Daly et al. 2018), recent genetic analyses have been able to identify several distinct mtDNA lineages or haplogroups (A, B, C, D, F, and G) in domestic goats (Luikart et al. 2001; Joshi et al. 2004; Sardina et al. 2006; Naderi et al. 2007, 2008). According to Naderi et al. (2008), haplogroup A, which accounts for nearly 90% of all domestic goat haplotypes and is globally widespread, may represent the evolutionarily ancestral lineage. This is further supported by its high haplotype diversity, which suggests an early divergence during the domestication process (Colli et al. 2015). Haplogroup B is found only in eastern and southern Asia, Africa, and Greece, while haplogroup C is found across all of Asia and Europe. Similarly, haplogroup D is present throughout Asia and in northern Europe. Haplotypes from Sicily represent haplogroup F, while those from the Middle East and Northern Africa, near the Fertile Crescent, where Türkiye is also an adjacent region, correspond to haplogroup G. Aside from these known domestic goat lineages, ancient goat samples from Southern France have the A and C haplotypes as well, and it has also been determined that ancient wild goat specimens excavated from the Direkli Cave in the Taurus Mountains of southern Türkiye may belong to a different lineage, which is referred to as haplogroup T (Fernández et al. 2006; Daly et al. 2018, 2022). In addition, it has been determined that six different haplogroups observed in domestic goats were also found in wild goats in different geographical regions (A in Eastern Anatolia, B in Northern Iran and Eastern Anatolia, C in the Zagros Mountains, D and G in Northern Iran, F in areas close to the North Caucasus) at lower frequencies than in domestic goats (Colli et al. 2015). Haplotype A, which is common among domestic goats, also appears in wild goat populations in Eastern Anatolia. Its absence in wild goats from the Zagros Mountains suggests that domestic goats may have been first domesticated in Anatolia rather than the Zagros region. This observation supports the hypothesis that Anatolia could have been a key centre of diversification, providing the genetic foundation for the domestication and spread of goats (Bruford et al. 2003; Çınar Kul and Ertugrul 2011; Colli et al. 2015). Overall, these findings highlight that goat domestication likely involved multiple regions within the Fertile Crescent and surrounding areas, with Anatolia, the Zagros Mountains, and parts of Iran playing key roles (Luikart et al. 2001). The presence of these haplotypes in both domestic and wild goats, albeit with varying frequencies, underscores the interconnectedness of these regions in the evolutionary history of the domestic goat (Colli et al. 2015).

Additionally, studies conducted to uncover the genetic makeup of livestock may also provide a way to understand the genetic diversity that could have been lost due to genetic drift as populations moved away from the centre of diversification or domestication (Meadows et al. 2007). In this regard, Eastern Anatolia and its neighboring regions such as Iraq, located within the Fertile Crescent, are particularly significant as the centre of goat domestication (Zeder and Hesse 2000; Zeder 2008). Although studies on the evolutionary relationships within the genus *Capra*, based on both mitochondrial and nuclear DNA, have yielded conflicting phylogenies, the results generally reveal an evolutionary pattern that aligns closely with the current geographical distributions of both domesticated and wild species within this genus (Manceau et al. 1999; Pidancier et al. 2006; Kazanskaya et al. 2007). In addition to these studies, large sample size studies based on genomic approaches have recently been conducted to obtain a global perspective on the phylogeography and molecular genetic diversity of *C. hircus* . Accordingly, three main gene pools corresponding to domestic goats in Europe, Africa, and Western Asia after domestication have been identified in the Fertile Crescent, as well as regional gene pools reflecting the main migration routes (Colli et al. 2015, 2018). The phylogeography of the genus *Capra*, especially of *C. hircus* , is consistent with the phylogeographic scenario of the origin and adaptive expansion of the subfamily Caprinae to which it belongs. Molecular studies and phylogeographic analyses imply that the common ancestor of the Caprinae subfamily spread from Africa to Asia and Europe during the Miocene, followed by rapid evolutionary diversification within the subfamily in these continents (Randi et al. 1991; Lalueza‐Fox et al. 2005; Ropiquet and Hassanin 2005a, 2005b; Hassanin et al. 2012). Although the domestication of *C. hircus* occurred approximately 10,000–11,000 years ago, mitochondrial DNA studies provided evidence that the most recent common ancestor of *C. hircus* lineages may have diversified much earlier, around 840,000 to 103,000 years ago (Luikart et al. 2001; Chen et al. 2005; Sultana et al. 2003; Joshi et al. 2004; Nomura et al. 2013; Colli et al. 2015).

Molecular studies using partial mitochondrial DNA sequences or microsatellites conducted on Türkiye's domestic goat breeds and wild goats provided valuable insights into their evolutionary history and domestication processes. Mitochondrial DNA sequence analysis has revealed that some domestic goat breeds in the Anatolian part of Türkiye (Angora, Kilis, Honamlı, Hair and Norduz) carry haplotypes A, D, and G. This suggests that Anatolia was a potential center for the domestication of goats, highlighting the role of the region in the origin of modern domestic goats (Çınar Kul and Ertugrul 2011). Another study on both wild and domestic goats in Türkiye highlighted a clear genetic differentiation between eastern and western wild goat populations. Domestic goats clustered more closely with eastern wild goat populations, suggesting that domestication may have occurred in eastern Türkiye, reinforcing the idea of Türkiye being a crucial region for goat domestication (Balkız 2002). A recent study based on morphology and mitochondrial DNA D‐loop sequencing revealed significant genetic and morphological diversity between bezoar populations in a narrow area and suggested that the influence of introgressive hybridization and distinct gene pools may have acted in shaping the observed traits (Şentürk et al. 2023).

Given the regional significance of the Fertile Crescent, a key area in the early stages of goat domestication, the current study focused on presenting novel mitogenome sequences of domestic goat breeds ( *C. hircus* ), wild goat ( *C. aegagrus* ) and chamois ( *R. rupicapra* ) species from Türkiye and Iraq. Thus, the study aimed to present phylogenetic and phylogeographic insights into the genus *Capra* by using complete mitochondrial genome sequences. This effort is crucial for deepening our understanding of the domestication process, as well as the evolutionary history and distribution of goats in this critical geographic region.

## 2 Materials and Methods

### 2.1 Ethics Statement, Experimental Design, and Mitogenomic Data

**FIGURE 1:**
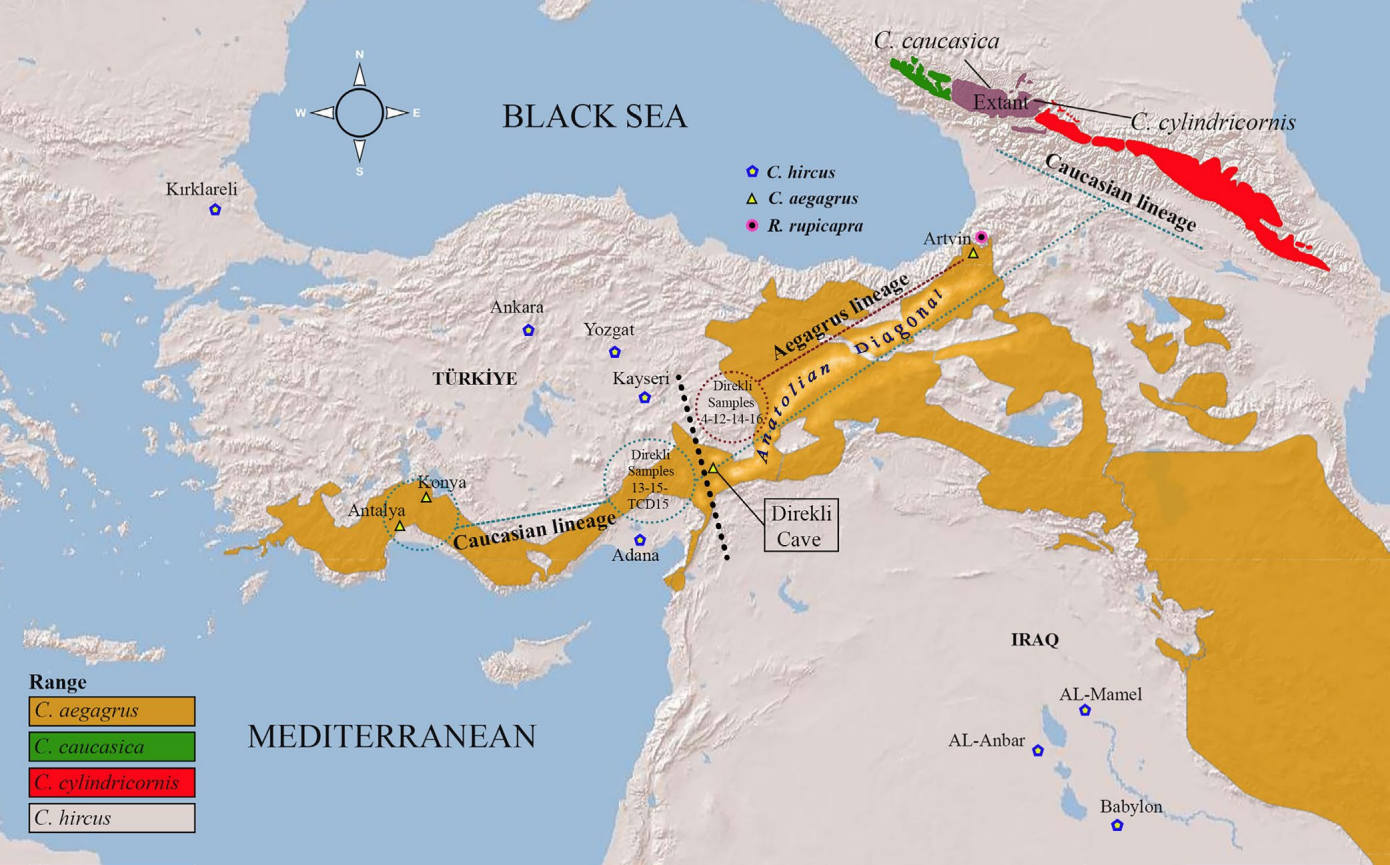
Geographic locations of goat samples collected from Türkiye and Iraq.

All study samples were collected under a permit issued by the Ministry of Agriculture and Forestry, Republic of Türkiye, with the date and number as follows: 08.08.2023/E‐21264211‐288.04‐10822834. Experimental studies were conducted under the auspices of the Ethics Committee of Erciyes University (Kayseri, Türkiye) with the date and number as follows: 05.04.2023/23‐061‐04. Tissue samples from domestic goats were obtained post‐mortem at authorized slaughterhouses, ensuring no animals were harmed specifically for research. Samples from wild goats and chamois, provided by Türkiye's Nature Conservation Directorate, originated from animals found dead in the field. All procedures adhered to ethical guidelines for the use of animals in research. Experimental studies utilized muscle tissue samples from 26 domestic goats representing various breeds—Angora, Saanen, and Hair goats from Türkiye, and Local, Shami, and Cyprus goats from Iraq—as well as samples from three wild goats and one chamois from Türkiye (see Figure 1 and Table 1). The experimental stages (genomic DNA extraction, amplification of the complete mitogenome, and next‐generation sequencing) and bioinformatic analyses (assembly, annotation and analyses of mitogenomic sequences) were conducted at the Genome and Stem Cell Center (GenKök) in Erciyes University, Kayseri, Türkiye.

**TABLE 1:** Information about the goat samples used in this study.

Voucher code	Accession number	Species/Breed	Mitogenome length	Location
CaHiTR1	PV138197	*C. hircus* (Turkish Saanen goat)	16,640	Kırklareli, Türkiye
CaHiTR2	PV138198	*C. hircus* (Hair goat)	16,643	Adana, Türkiye
CaHiTR3	PV138195	*C. hircus* (Hair goat)	16,640	Adana, Türkiye
CaHiTR4	PV138196	*C. hircus* (Hair goat)	16,641	Adana, Türkiye
CaHiTR5	PV138206	*C. hircus* (Turkish Saanen goat)	16,640	Yozgat, Türkiye
CaHiTR6	PV138205	*C. hircus* (Angora goat)	16,644	Ankara, Türkiye
CaHiTR7	PV138214	*C. hircus* (Hair goat)	16,651	Kayseri, Türkiye
CaHiTR8	PV138213	*C. hircus* (Kilis goat)	16,649	Kayseri, Türkiye
CahiIRQ1	PV138189	*C. hircus* (Local goat)	16,642	Samarra, Al‐Mamel, Iraq
CahiIRQ2	PV138190	*C. hircus* (Unknown)	16,641	Hillah, Babylon, Iraq
CaHiIRQ3	PV138211	*C. hircus* (Local goat)	16,640	Hit, AL‐Anbar, Iraq
CaHiIRQ4	PV138191	*C. hircus* (Cyprus goat)	16,640	Hit, AL‐Anbar, Iraq
CaHiIRQ5	PV138193	*C. hircus* (Shami goat)	16,640	Hit, AL‐Anbar, Iraq
CaHiIRQ6	PV138194	*C. hircus* (Cyprus goat)	16,640	Hit, AL‐Anbar, Iraq
CaHiIRQ7	PV138210	*C. hircus* (Shami goat)	16,639	Hit, AL‐Anbar, Iraq
CaHiIRQ8	PV138209	*C. hircus* (Local goat)	16,641	Jamiah, Samarra, Iraq
CaHiIRQ9	PV138192	*C. hircus* (Local goat)	16,640	Jamiah, Samarra, Iraq
CaHiIRQ10	PV138202	*C. hircus* (Local goat)	16,641	Hit, AL‐Anbar, Iraq
CaHiIRQ11	PV138200	*C. hircus* (Unknown)	16,640	Hillah, Babylon, Iraq
CaHiIRQ12	PV138199	*C. hircus* (Local goat)	16,640	Hillah, Babylon, Iraq
CaHiIRQ13	PV138208	*C. hircus* (Cyprus goat)	16,640	Hadi, Samarra, Iraq
CaHiIRQ14	PV138201	*C. hircus* (Unknown)	16,639	Hillah, Babylon, Iraq
CaHiIRQ15	PV138204	*C. hircus* (Unknown)	16,641	Hillah, Babylon, Iraq
CaHiIRQ16	PV138207	*C. hircus* (Unknown)	16,640	Hillah, Babylon, Iraq
CaHiIRQ17	PV138212	*C. hircus* (Unknown)	16,639	Hillah, Babylon, Iraq
CaHiIRQ18	PV138203	*C. hircus* (Unknown)	16,642	Hillah, Babylon, Iraq
CaAeTR1	PV138188	*Capra aegagrus* (Wild goat, Aegagrus lineage)	16,639	Yusufeli, Artvin, Türkiye
CaCaTR1	PV138215	*Capra aegagrus* (Wild goat, Caucasian lineage)	16,623	Akseki, Antalya, Türkiye
CaCaTR2	PV138216	*Capra aegagrus* (Wild goat, Caucasian lineage)	16,622	Derebucak, Konya, Türkiye
RuRuTR1	PV138217	*Rupicapra rupicapra* (Northern Chamois)	16,434	Artvin, Türkiye

Genomic DNA from hair and muscle tissues was extracted using the DNeasy Blood and Tissue Kit (QIAGEN) according to the manufacturer's instructions. The complete mitogenomes were amplified using Long‐Range PCR, including specific primer pairs and the NEB LongAmp Taq 2 × Master Mix from New England Biolabs (see for details İbiş 2020 and Şeker 2024). Paired‐end sequencing of the complete mitogenomes was achieved by a Nextera XT DNA Library Prep Kit (FC‐131–1096, Illumina, San Diego, USA) and a Nextera XT DNA Library Preparation Index Kit v2 Set A (FC‐ 131–2001, Illumina, San Diego, USA), followed by sequencing of the prepared libraries on an Illumina MiSeq instrument at the Genome and Stem Cell Center—GenKok (Erciyes University, Kayseri, Türkiye). The raw fastq files were imported as paired reads into Geneious Prime 2024.0 software (https://www.geneious.com) and low‐quality reads (shorter than 50 bases and Q‐score less than 25) were then trimmed using the BBDuk v38.84 plugin (https://github.com/BioInfoTools/BBMap/blob/master/sh/bbduk.sh). The goat mitogenomes were assembled using the Geneious Mapper Algorithm, with the mitogenome of *Capra hircus* (GenBank accession number: NC_005044) as the reference. The assembly was performed with the highest sensitivity/medium setting and fine‐tuned by iterating up to 25 times. Additionally, the GetOrganelle toolkit (Jin et al. 2020) with default parameters was used to *de‐novo* assemble the reads into mitogenomes. The BBMap tool (with the Normal Sensitivity parameter) in Geneious Prime was used to remap the filtered and trimmed sequence reads to the *de‐novo* contigs, ensuring coverage and completeness. The gene boundaries of each goat mitogenome were analyzed using the MITOS2 web server (Donath et al. 2019) and manually edited as needed.

### 2.2 Phylogenetic Analyses and Evolutionary Dating

A data set including mitogenome sequences was compiled from the samples obtained in this study as well as from other taxa of the Caprinae downloaded from NCBI's GenBank database (Supporting Information File 1). A multiple sequence alignment was generated using the MAFFT version 7.450 (Katoh et al. 2002) plugin in Geneious Prime 2024.0. Gblocks v0.91b with default parameters (Castresana 2000) was employed to mask the ambiguously aligned regions of the mitogenome dataset. A model‐based Maximum Likelihood (ML) phylogenetic analysis was conducted in MEGA X (Kumar et al. 2018). Using the Bayesian Information Criterion (BIC) in jModelTest 2.1.10 (Darriba et al. 2012), the GTR + G + I (General Time Reversible + Gamma + Invariant sites) model was determined to be the best‐fitting nucleotide substitution model for the dataset. Nonparametric bootstrap statistics with 1000 replicates were used to test the robustness of the ML tree topology.

Bayesian Inference (BI) analysis was also executed using BEAST v1.8.0 (Drummond et al. 2012) and the GTR + G + I model of nucleotide substitution. The Yule model of speciation was selected as the tree prior, and a strict molecular clock model was used for the mitogenomic DNA data set, which is inherited as a single linkage group. Three independent MCMCMC (Metropolis‐coupled Markov Chain Monte Carlo) runs were performed, each for 10,000,000 generations with a sampling frequency of every 1000 generations. The three resulting logfiles produced by the three independent runs were combined using LogCombiner v1.8.0 (Drummond et al. 2012). Tracer v1.7.2 (Rambaut et al. 2018) was used to determine whether the effective sample sizes for the inferred tree, clock model, and other model parameters were sufficient (Effective Sample Sizes > 200) after combining the three logfiles. The three tree files, each containing 10,000 trees, were united into a single file with LogCombiner v1.8.0 (Drummond et al. 2012). TreeAnnotator v1.8.0 was executed to summarize the united tree file containing 30,000 trees by excluding the first 10% (3000 trees) of the sampled trees as burn‐in, and a 50% majority‐rule consensus tree was created to calculate the Bayesian Posterior Probabilities (BPPs) and divergence times of the tree nodes. The initial divergence date within Caprinae (9.2 ± 1.8 million years ago, Mya), as determined by Hassanin et al. (2012), was used as a calibration point in the molecular dating analysis to estimate the diversification events within the subfamily Caprinae investigated in the current study. The Interactive Tree of Life (iTOL) v5 online tool (Letunic and Bork 2024) was used to visualize and edit the phylogenetic tree created by the BI analysis. In addition to the ML and BI analyses, a median‐joining network (Bft et al. 1999) was constructed using PopART 1.7 (Leigh et al. 2015) to illustrate the evolutionary relationships among both main haplogroups of domestic goat and wild goat species. Haplotype diversity and nucleotide diversity, along with other sequence statistics, were estimated using DNAsp v6.12.03 (Rozas et al. 2017). Finally, genetic distance estimations were calculated using the Kimura‐2 parameter (K2P) model with 1000 bootstrap replicates executed in MEGA X (Kumar et al. 2018).

## 3 Results

Each of the mitogenomes of domestic and wild goat species, and one chamois, obtained in this study consisted of 13 protein‐coding, 2 ribosomal RNA, and 22 transfer RNA genes, a control region (also known as the D‐loop), and a putative origin of replication of the light strand (O_L_). Arrangement and organization of the mitogenomes were similar to other mammals and contained 16,644 bp for domestic goat (CaHiTR6), 16,622 bp for wild goat (CaCaTR2), and 16,434 bp for northern chamois (RuRuTR1). Genome size variation in the mitogenomes of each species was due to D‐loop length differences. The nucleotide content of the mitogenomes was as follows: A = 33.52%, T = 27.30%, C = 26%, and G = 13.10% for domestic goat; A = 33.50%, T = 27.20%, C = 26.20%, and G = 13.10% for wild goat; A = 33.30%, T = 26.10%, C = 27.1%, and G = 13.50% for northern chamois.

A total of 25 unique haplotypes were detected based on variation in the complete mitochondrial genome sequences obtained from the 26 domestic goat samples collected in eight different localities across Türkiye and Iraq. Three novel haplotypes were detected in wild goat, and one novel haplotype was detected in the northern chamois sample (Table 2, and Supporting Information File 1). Genetic diversity statistics for domestic and wild goat samples from Türkiye and Iraq, along with the main haplogroups in domestic goats, are shown in Table 3. Multiple sequence alignment of all domestic goat samples from Türkiye and Iraq yielded 161 polymorphic sites, of which 29 were parsimony‐informative. The estimated average transition to transversion ratio was 14.98. Haplotype diversity was high in domestic goat breeds in both Türkiye and Iraq. Although the number of Turkish samples was fewer than the Iraq samples, the nucleotide diversity and average number of nucleotide differences observed in domestic goat samples from Türkiye were higher than those from Iraq (Table 3). Based on the genetic distance values calculated using the Kimura 2‐parameter model, the Hair goat breed in Türkiye was found to be the genetically most distant from the other domestic goat breeds (Table 4). Genetic distance values among wild goat species are provided in Table 5.

**TABLE 2:** List of haplotypes of domestic goat samples from Türkiye and Iraq, along with those of wild goat and chamois species from Türkiye.

H	F	Voucher number	Locality	H.G.	A.N.
**Domestic goat**					
1	1	CaHiTR6	Ankara, Türkiye	A	PV138205
2	1	CaHiTR7	Kayseri, Türkiye	G	PV138214
3	1	CaHiTR8	Kayseri, Türkiye	A	PV138213
4	1	CaHiTR2	Adana, Türkiye	A	PV138198
5	1	CaHiTR1	Kırklareli, Türkiye	A	PV138197
6	2	CaHiTR4 and CaHiTR3^a^	Adana, Türkiye	A	PV138195
7	1	CaHiTR5	Yozgat, Türkiye	A	PV138206
8	1	CaHiIRQ12	Hillah, Babylon, Iraq	A	PV138199
9	1	CaHiIRQ14	Hillah, Babylon, Iraq	A	PV138201
10	1	CaHiIRQ4	Hit, AL‐Anbar, Iraq	A	PV138191
11	1	CaHiIRQ9	Jamiah, Samarra, Iraq	A	PV138192
12	1	CaHiIRQ18	Hillah, Babylon, Iraq	A	PV138203
13	1	CaHiIRQ15	Hillah, Babylon, Iraq	A	PV138204
14	1	CaHiIRQ10	Hit, AL‐Anbar, Iraq	A	PV138202
15	1	CaHiIRQ8	Jamiah, Samarra, Iraq	A	PV138209
16	2	CaHiIRQ5 and CaHiIRQ6	Hit, AL‐Anbar, Iraq	A	PV138193, PV138194
17	1	CaHiIRQ16	Hillah, Babylon, Iraq	A	PV138207
18	1	CaHiIRQ11	Hillah, Babylon, Iraq	A	PV138200
19	1	CaHiIRQ3	Hit, AL‐Anbar, Iraq	A	PV138211
20	1	CaHiIRQ17	Hillah, Babylon, Iraq	A	PV138212
21	1	CaHiIRQ7	Hit, AL‐Anbar, Iraq	A	PV138210
22	1	CaHiIRQ1	Samarra, AL‐Mamel, Iraq	A	PV138189
23	1	CaHiIRQ2	Hillah, Babylon, Iraq	A	PV138190
24	1	CaHiIRQ13	Hadi, Samarra, Iraq	A	PV138208
**Wild goat**					
	1	CaCaTR1	Akseki, Antalya, Turkiye		PV138215
	1	CaCaTR2	Derebucak, Konya, Turkiye		PV138216
	1	CaAeTR1	Yusufeli, Artvin, Turkiye		PV138188
**Northern chamois**					
	1	RuRuTR1	Artvin, Türkiye		PV138217

Abbreviations: A.N., GenBank accession number; F, frequency; H, Haplotype number; H.G., domestic goat haplogroup assignment.

^a^ These two sequences represent different haplotypes based on complete mitogenome data; however, when ambiguously aligned regions of the mitogenome dataset are filtered using Gblocks, they constitute a single haplotype with the same nucleotide sequence.

**TABLE 3:** Genetic diversity statistics for domestic goat samples from Türkiye and Iraq, along with those of the main haplogroups in *C. hircus* .

	N	H	S	SV	PI	HD	*π*	k
Türkiye	8	7	74	64	10	0.964 ± 0.077	0.00133 ± 0.00038	20.500
Iraq	18	17	92	74	18	0.993 ± 0.021	0.00081 ± 0.00008	12.529
All	26	24	161	132	29	0.994 ± 0.013	0.00098 ± 0.00015	15.052
*Haplogroups*								
A	56	54	268	222	46	0.999 ± 0.004	0.00080 ± 0.00005	12,270
B	1	1	n.c.	0	0	n.c.	n.c.	n.c.
C	2	2	11	11	0	1.000 ± 0.500	0.00071 ± 0.00036	11
D	2	1	0	0	0	0.000 ± 0.000	0.00000 ± 0.00000	0.00
G	6	6	38	37	1	1000 ± 0.096	0.00084 ± 0.00013	12,867
All	67	64	441	272	169	0.999 ± 0.003	0.00151 ± 0.00023	23,279

Abbreviations: π, nucleotide diversity with standard deviation; H, number of haplotypes; HD, haplotype diversity with standard deviation; k, average number of nucleotide differences; N, sample size; n.c., not computed; PI, parsimony‐informative sites; S, number of segregating sites; SV, singleton variable sites.

**TABLE 4:** Genetic distances based on the Kimura 2‐parameter model among domestic goat breeds in Türkiye.

	Angora	Hair	Kilis	Saanen
Angora		0.000180	0.000272	0.000165
Hair	0.001139		0.000269	0.000179
Kilis	0.001171	0.001709		0.000271
Saanen	0.000650	0.001326	0.001334	

**TABLE 5:** Genetic distances based on the Kimura 2‐parameter model among wild goats in Türkiye.

	1	2	3	4	5	6
1. Artvin		0.00059	0.00133	0.00132	0.00130	0.00130
2. Direkli Cave 1 (Samples 4, 12, 14, and 16) Daly et al. 2022)	0.00677		0.00127	0.00126	0.00127	0.00127
3. Konya‐Antalya	0.02585	0.02549		0.00038	0.00099	0.00096
4. Direkli Cave 2 (Samples 13, 15, and TCD‐15) Daly et al. 2022)	0.02547	0.02507	0.00250		0.00098	0.00094
5. *C. caucasica*	0.02531	0.02485	0.01456	0.01422		0.00078
6. *C. cylindricornis*	0.02531	0.02479	0.01409	0.01377	0.00911	

**FIGURE 2:**
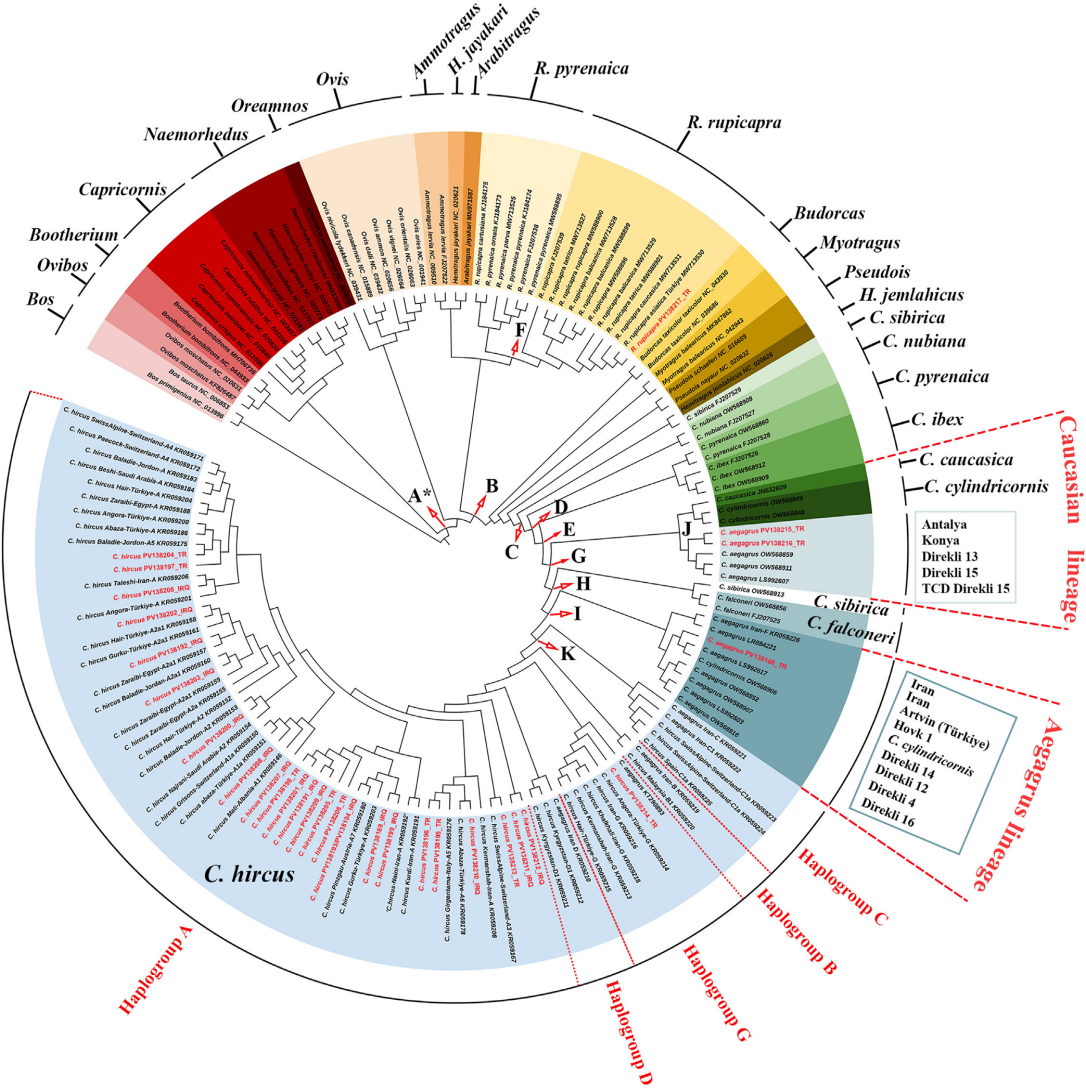
Phylogenetic tree and divergence dating results based on Bayesian inference analyses using BEAST v1.8.0. A‐K on the nodes of the circular phylogenetic tree indicate the evolutionary divergence times among the Caprinae lineages presented in Table 6. The ML phylogenetic tree exhibited the same topology except the position of the *Ovis* clade (Supporting Information File 2). For specific node ages, posterior probability values, 95% highest posterior densities (HPDs) and also K‐2P distances for the main nodes, see Table 6.

The ML and BI analyses produced phylogenetic trees with similar topologies, except for the position of the clade containing species in the genus *Ovis* (Figure 2 and Supporting Information File 2). According to the topology of both phylogenetic trees, species‐based separations among the Caprinae phylogeny were robustly resolved except for some small deviations. In the domestic goat clade, which occupies the innermost branch of the phylogenetic tree, five wild goat samples ( *C. aegagrus* ) from Iran were clustered with the domestic goat samples. Apart from these exceptional cases, a complex phylogeny was observed in this clade, intertwining samples representing different domestic goat breeds in Türkiye (Hair, Angora, Kilis, and Saanen) and different goat breeds in Iraq (Local, Cyprus, and Shami). There were samples representing previously identified haplogroups A, B, C, D, and G obtained from GenBank in the domestic goat clade. A Hair goat sample from Kayseri (Türkiye) was clustered with goat samples from Türkiye and Iraq obtained from GenBank, all representing haplogroup G. In contrast, all other domestic goat samples generated in this study clustered with the GenBank samples, forming haplogroup A. One Angora goat sample from Ankara (Türkiye) was clustered with the Local goat samples from Samarra and Hilla (Iraq). Two Saanen goat samples, one from Kırklareli and one from Yozgat (Türkiye) were grouped with three Hair goat samples (one from Adana, Türkiye, and two from Hilla, Iraq), two Shami goat samples, one Cyprus goat sample from Al‐Anbar (Iraq), and *C. hircus* samples obtained from GenBank. Apart from these, two Hair goat samples from Adana (Türkiye) were grouped with the two *C. hircus* samples obtained from GenBank. One Kilis goat sample from Kayseri (Türkiye) was clustered with one Local goat sample from Samarra (Iraq).

On the other hand, the Turkish wild goats ( *C. aegagrus* ) were divided into two distinct lineages (an Aegagrus lineage and a Caucasian lineage) in the phylogenetic trees, which indicated a paraphyletic relationship for this species. The Aegagrus lineage was sister to the lineage comprising the domestic goat samples, and it included one *C. aegagrus* sample from Artvin (northeast of Türkiye) generated in this study along with the previously reported four ancient samples of *C. aegagrus* (OW568852‐Direkli 14, OW568907‐Direkli 12, OW568916‐Direkli 16, LS992603‐Direkli 4) from Direkli Cave (in the Taurus mountains from southeastern Türkiye), one medieval sample of *C. cylindricornis* (OW568906‐East Caucasian tur) from Georgia, one ancient sample of *C. aegagrus* from Hovk 1 Cave in Armenia (LS992617‐Hovk1), and two wild goat samples (one was ancient) from Iran (LR88422‐ Ganjdareh35, KR059226). The Caucasian lineage was positioned as a distinct haplogroup but separated from specimens of *C. sibirica* and *C. falconeri* . In the Caucasian lineage, samples from Konya and Antalya in the southern part of Türkiye were clustered with three ancient samples of *C. aegagrus* from Direkli Cave (OW568859‐Direkli 13, OW568911‐Direkli 15, LS992607‐TCD Direkli 15). This lineage of wild goats was grouped with a haplogroup containing two *C. cylindricornis* samples and one *C. caucasica* sample from GenBank. Two other species, *C. pyrenaica* and *C. ibex* , were positioned basal to the aforementioned lineages in the ML and BI trees. The chamois sample from Türkiye, whose complete mitogenome sequence was obtained in the current study, was included in the *R. rupicapra* lineage, which was divided into three sublineages as expected (Figure 2). Other genera included in the Caprinae phylogeny were clustered in the following order from inside to outside towards the base of the ML phylogenetic tree: *Hemitragus*, *Psedois*, *Ammotragus*, *Arabitragus*, *Hemitragus jayakari* , *Ovis*, *Oreamnos*, *Budorcas*, *Myotragus*, *Rupicapra*, *Bootherium*, *Ovibos*, *Capricornis*, and *Naemorhedus* (Figure 2 and Supporting Information File 2).

Divergence time analysis based on Bayesian Inference (BI) estimated that the evolutionary diversification within Caprinae started about 8.18 Mya ago (Node A; 95% Highest Probability Density, HPD: 4.33–11.99 Mya and BPP: 1). The divergence dating and posterior probability results are presented in Figure 2 and Table 6. The *Capra/Rupicapra* split was found to have occurred approximately 6.91 Mya ago (Node B; 95% HPD: 3.63–10.07 Mya and BPP: 0.78). The *R. rupicapra* / *R. pyrenaica* split within *Rupicapra* was calculated to have occurred approximately 1.5 Mya (Node F; 95% HPD: 0.77–2.19 Mya and BPP: 1). When focusing on the evolutionary history of the genus *Capra*, it was revealed that the diversification events within this genus began approximately 3.22 Mya (Node C; 95% HPD: 1.69–4.72 Mya and BPP: 1). The first group at the base of the phylogenetic tree consisted of haplotypes belonging to *C. sibirica* and *H. jemlahicus* (Node C). *C. nubiana* , which is distributed in northern Africa and the Middle East, was the second differentiated species in the BI tree (Node D). The onset of evolutionary differentiation of this species dated back approximately 1.75 Mya. Apart from this species, two wild goat species localized in proximate geographical areas, the Iberian ibex *C. pyrenaica* and the Alpine ibex *C. ibex* , were found to have differentiated approximately 1.54 Mya (Node E). As in the ML tree, the Caucasian lineage that included living and ancient samples of wild goats from Türkiye, *C. cylindricornis* and *C. caucasica* was estimated to have started to diverge approximately 1.16 Mya (Node G). As for the age of the beginning of differentiation within this main lineage (Wild goats from Türkiye/ *C. cylindricornis* + *C. caucasica* ), this was dated at 0.64 Mya (Node J). The evolutionary divergence of the lineage consisting of the *C. sibirica* and the *C. falconeri* haplotypes was found to have begun approximately 1.11 Mya (Node H). The time of evolutionary divergence between the Aegagrus lineage and the lineage consisting of domestic goats, *C. hircus* , was determined to correspond to approximately 0.89 Mya (Node I). It was determined that the beginning of the evolutionary differentiation within the domestic goat lineage was approximately 0.29 Mya (Node K) (Figure 2).

**TABLE 6:** Estimates of the evolutionary divergence times within the subfamily Caprinae based on Bayesian inference analysis.

Nodes		Node ages (Mya)	%95 HPD (Mya)	BPP	K‐2P (%)
A	Within Caprinae	8.18	4.33–11.99	1	—
B	*Capra*/*Rupicapra*	6.91	3.63–10.07	0.78	11.2 ± 0.003
C	Within *Capra* ( *C. sibirica* + *H. jemlahicus/……*)	3.22	1.69–4.72	1	6.11 ± 0.0017
D	*C. nubiana*	1.75	0.93–2.57	1	3.5 ± 0.0014
E	*C. pyrenaica* / *C. ibex*	1.54	0.80–2.24	1	1.38 ± 0.0008
F	*R. rupicapra* / *R. pyrenaica*	1.50	0.77–2.19	1	3.29 ± 0.0013
G	*C. aegagrus* + *C. cylindricornis* + *C. caucasica* /*……*	1.16	0.60–1.69	1	2.53 ± 0.0011
H	*C. sibirica* + *C. falconeri* */……*	1.11	0.59–1.63	0.99	2.5 ± 0.0011
I	*C. aegagrus* / *C. hircus*	0.89	0.47–1.31	1	2 ± 0.0010
J	Wild goats from Türkiye/ *C. cylindricornis* + *C. caucasica*	0.64	0.33–0.94	1	1.41 ± 0.0009
K	Within *C. hircus*	0.29	0.15–0.42	1	—

*Note:* The nodes (A–K) in the table are also shown in Figure 2.

Abbreviations: BPP, Bayesian posterior probability; HPD, high posterior density; K‐2P, Kimura‐2 parameter distance; Mya, millions of years ago.

**FIGURE 3:**
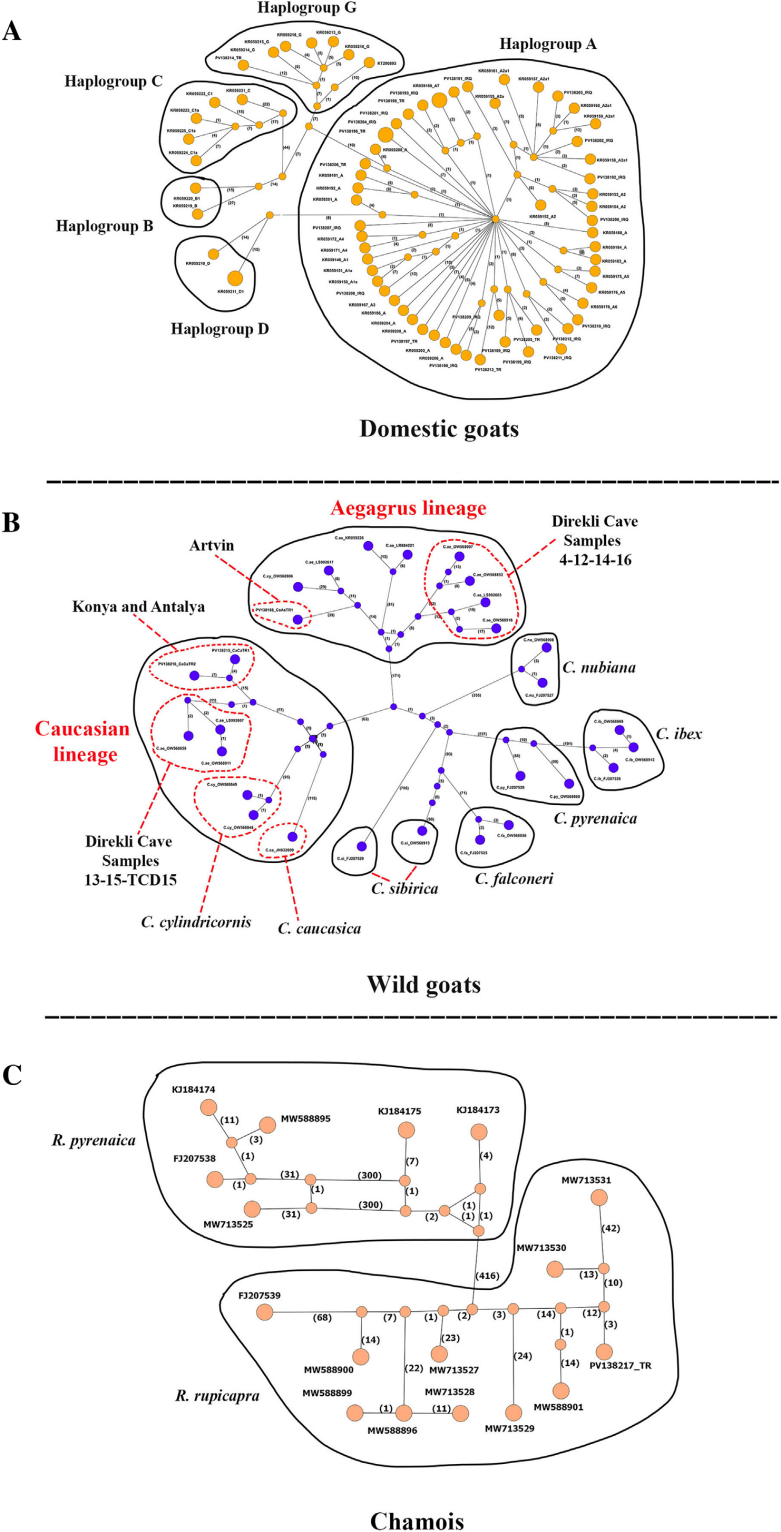
The median‐joining haplotype network reconstructed based on complete mitochondrial genome sequences illustrates the relationships among the main haplogroups (haplogroups A, B, C, D, and G) in domestic goats (A) and species relationships among wild goats, and chamois (B and C, respectively). Shared haplotypes in haplogroup A and haplogroup D (Haplotypes 6 and 56) are shown in different colors. The circles are proportional to the sample size.

The results of the median‐joining haplotype network analysis, which was performed to display the evolutionary relationships among the major haplogroups of domestic goats, revealed a structure that was consistent with the topology of the phylogenetic trees obtained from the ML and BI analyses (Figure 3). A total of 70 haplotypes were identified from 73 goat samples (68 domestic and 5 wild goat samples) in the network analysis (Figure 3A and Supporting Information File 1). In domestic goats, the network revealed five major haplogroups consistent with the phylogenetic tree topology, with the minimum number of mutational steps ranging from 23 to 95. These were supported by bootstrap values of 100% and Bayesian posterior probabilities (BPPs) of 1, indicating strong concordance between the network structure and phylogenetic inferences (Figure 3A). Among these, haplotypes PV138204, KR059180, and KR059211 each had a frequency of 2, while all other haplotypes were found at a frequency of 1. Of the total haplotypes, 54 belonged to haplogroup A (approximately 77.14% of the total), 2 to haplogroup B and D, 5 to haplogroups C, and 7 to haplogroup G. When compared with haplogroup A, which contains the most haplotypes, and other haplogroups, haplogroup A was the closest to haplogroup D (8 mutation steps). However, the maximum number of mutations was observed between haplogroup A and haplogroup C (61 mutation steps). On the other hand, 45 haplotypes were determined to belong to the other *Capra* and *Rupicapra* haplogroups (Figure 3B,C). The number of mutations between the Aegagrus lineage (with 9 haplotypes) and the Caucasian lineage (with 9 haplotypes) was 234. Additionally, there were 416 mutations between the two chamois species ( *R. rupicapra* / *R. pyrenaica* ). Genetic distances calculated among the main nodes in the phylogenetic trees reconstructed in the ML and BI analyses using the Kimura‐2 parameter model are presented in Table 6. Accordingly, the genetic distance between the Aegagrus lineage and the domestic goat lineage ( *C. hircus* ) was approximately 2%, aligning with the estimated evolutionary divergence time of 0.89 Mya, based on a mitochondrial genome mutation rate of 2% per million years. Similarly, genetic distance values between the other major clades were also consistent with their respective evolutionary divergence times.

## 4 Discussion

This study provided new mitogenome data for various domestic goat breeds from Türkiye and Iraq, as well as for two wild goat species ( *C. aegagrus* and *R. rupicapra* ) from Türkiye. It also included an assessment of genetic diversity, along with phylogenetic and phylogeographic inferences based on the mitogenome sequences obtained for these species. In previous studies based on partial sequencing of some regions of the mitochondrial genome and microsatellites (Çınar Kul and Ertugrul 2011; Ağaoğlu and Ertuğrul 2012), it was determined that Turkish goats have high levels of genetic diversity and no genetic bottleneck, along with the presence of haplotypes A, D, and G in some domestic goat breeds in Türkiye. Based on variation observed across the entire mitochondrial genome, the current study determined that most of the new mitogenomes sequenced from domestic goats belong to haplogroups A and G and revealed high haplotype and nucleotide diversity in domestic goats from Türkiye, thereby confirming the high level of genetic diversity reported in previous studies (Çınar Kul and Ertugrul 2011; Ağaoğlu and Ertuğrul 2012). Genetic diversity is expected to decrease with distance from the center of domestication, as groups that have left the gene pool will carry a limited number of genotypes rather than representing all genotypes in the gene pool (Loftus et al. 1999; MacHugh and Bradley 2001). It was determined that the haplotype diversity and nucleotide diversity values observed in domestic goat breeds in Türkiye were almost at the same level as the values observed in the genomes of all domestic goats from different geographic areas evaluated within the scope of the study. Results such as the high level of genetic diversity observed highlight the importance of the area in the emergence of modern domestic goats, allowing Türkiye and nearby regions to be considered as a potential center for goat domestication. Additionally, the shared haplotype A between domestic and wild goats ( *C. aegagrus* ) in Anatolia is considered a significant indicator leading to a hypothesis suggesting that this region can be an important genetic center that contributed to the domestication and spread of goats (Colli et al. 2015). In this context, it is believed that the goat domestication process, which significantly contributed to the agricultural revolution during the Neolithic period, began around 10,000 years ago in the Fertile Crescent, including Anatolia (Zeder and Hesse 2000; Luikart et al. 2001; Naderi et al. 2007, 2008; Colli et al. 2015).

Within the framework of this widely accepted view, eastern Anatolia has been proposed as a potential starting point for goat domestication and as a genetic center for domestic goats worldwide (Naderi et al. 2008; Colli et al. 2015). An archaeological site of particular cultural significance in this region is Göbeklitepe, located in southeastern Türkiye and dated to approximately 9500 years ago (Peters et al. 1999; Schmidt 2000). While no direct genetic data (e.g., ancient goat DNA) are currently available from the site, the presence of wild animal motifs and its chronological proximity to the Neolithic transition suggest that Göbeklitepe may represent an important sociocultural context for future research on early animal domestication in the Fertile Crescent. In this broader context, the current study provides valuable mitogenomic data from domestic goats that contribute to characterizing the gene pool and demographic history of domestic goats. These data will support future efforts to investigate evolutionary events such as founder effects, genetic bottlenecks, or demographic expansions associated with goat domestication and global dispersal (FAO 2015).

Phylogenetic and phylogeographic analyses revealed that the phylogenetic structure of various domestic goat breeds in Türkiye and Iraq was complex, and their phylogeographic structure was weak. This was expected, given that there are over a thousand known domestic goat breeds worldwide with extensive genetic mixing, resulting in low genetic differentiation among domestic goat breeds spread across relatively large areas (Luikart et al. 2001; FAO 2015). Another reason for this complex phylogeny and weak phylogeography may be the widespread distribution of haplogroup A across the globe (Naderi et al. 2007). A previous study based on partial D‐loop sequences disclosed that some domestic goat breeds in Türkiye (Angora, Kilis, Honamlı, Hair and Norduz) have haplotypes A, D, and G (Çınar Kul and Ertugrul 2011). The results of the present study confirmed the presence of only A and G haplotypes for complete mitogenome sequences of the domestic goat breeds from Türkiye and Iraq, while the D haplotype was not detected among our samples. The previous study had a much larger sample size, but only 453 bp of the D‐loop region was analyzed (Çınar Kul and Ertugrul 2011), whereas our study used a smaller sample size but analyzed the entire mitogenome sequence. The lack of haplotype D being detected may be due to either whole mitogenome sequencing producing more accurate results than partial sequencing, or the lack of overlap in the sample locations included in both studies. Another study based on microsatellite data showed that there is a clear genetic distinction between the Angora goat and other domestic goat breeds (Kilis, Honamlı, Hair, and Norduz goats) in Türkiye (Ağaoğlu and Ertuğrul 2012). In contrast, the Ankara goat was in the same clade with the Saanen and Hair goat breeds from Türkiye and the Shami, Local, and Cyprus breeds from Iraq in the phylogenetic trees obtained in the current study. Furthermore, when considering the K‐2P calculations among domestic goat breeds in Türkiye, it was observed that the Hair goat, rather than the Angora goat, was the genetically most distant breed from the others. Another finding that showed this genetic distinction was that a Hair goat sample from Kayseri was in haplogroup D. In addition, it was observed that the Ankara and Saanen breeds were the genetically closest breeds among all breeds examined from Türkiye, which was consistent with the results of the phylogenetic analyses. The results of two different studies on domestic and wild goats in Türkiye, based on Y chromosome and mitochondrial DNA D‐loop variations, suggested that the ancestors of domestic goats in Türkiye may have originated from wild goats in Eastern Anatolia (Balkız 2002; Çınar Kul et al. 2015). The Aegagrus lineage, which was sister to the domestic goat lineage in the phylogenetic tree (Figure 2), strongly supported this hypothesis related to the Eastern Anatolian origin. This lineage included a *C. aegagrus* sample from northeastern Türkiye as well as wild goat samples from Iran.

Previous studies using mitochondrial and nuclear DNA markers have similarly identified two main wild goat clades in phylogenetic analyses within the genus *Capra*. However, these markers produced discordant intrageneric phylogenies due to variable positions of wild goat species in the phylogenetic trees (Manceau et al. 1999; Pidancier et al. 2006; Kazanskaya et al. 2007). This discordance is thought to have arisen from introgression between ancestral populations of wild goat species prior to the radiation of the genus (Pidancier et al. 2006; Ropiquet and Hassanin 2006). According to the currently accepted phylogeny of the genus, *C. sibirica* is an outgroup. *C. nubiana* is closely related to *C. walie*, while *C. pyrenaica* is closely related to *C. ibex* . The wild goats in the Caucasus are represented by two species, *C. caucasica* and *C. cylindricornis* . *C. aegagrus* and *C. caucasica* are notably genetically close species. *C. falconeri* is separated from the other species of the genus by a small genetic difference (Kazanskaya et al. 2007; Hassanin et al. 2009; Zheng et al. 2020). In our study, the *Capra* phylogeny based on whole mitogenome sequences was consistent with the widely accepted current phylogeny (Pidancier et al. 2006; Kazanskaya et al. 2007). The presence of wild goat specimens from Iran in the *C. hircus* lineage (e.g., KR059221, KR059222, KR059219, KR059218, KR059213), along with the fact that the Aegagrus lineage (which also included a wild goat specimen from Artvin, Türkiye, sample ID: PV138188) was a sister clade to *C. hircus* , supports the wild origin of *C. hircus* (Balkız 2002; Çınar Kul et al. 2015; Colli et al. 2015). A novel wild goat lineage, potentially related to *C. caucasica* , was discovered in Direkli Cave in the Taurus Mountains of southeastern Türkiye through ancient genomics (Daly et al. 2022). This lineage, dated to 12,000 years ago, diverged from wild goat lineages in the Caucasus around 130,000 to 200,000 years ago and was identified as the sister group of *C. caucasica* and *C. cylindricornis* . While Daly et al. (2022) suggested this lineage was extinct, the phylogenetic analysis in this study grouped together the Direkli samples (Direkli 4 and 16) with a wild goat sample from Artvin in the northeastern part of Türkiye (PV138188). This formed the Aegagrus lineage in the current study. The Caucasian lineage, consisting of samples from Konya, Antalya (PV138215, and PV138216) and other Direkli Cave specimens (Direkli 13, 15, and TCD 15), formed a sister group to *C. caucasica* and *C. cylindricornis* . Interestingly, the ancient lineage suggested as a new lineage by Daly et al. (2022) was placed in the Aegagrus lineage at the base of the domestic goat lineage, distant from the Caucasian lineages. Moreover, we found a close genetic relationship between the new lineage of Daly et al. (2022) and the Artvin sample (with a K‐2P genetic distance of 0.67%) and a relatively high genetic distance with two Caucasian wild goat species (JN632609— *C. caucasica* , OW568849 and OW568848‐ *C. cylindricornis* ) (with a K‐2P genetic distance of 2.5%). According to Bradley and Baker (2001), species delimitation under the genetic species concept relies on genetic distances exceeding specific threshold values to delineate separate species. The low genetic distance observed here suggests that the new lineage and the Artvin sample likely belong to the same species, while the higher genetic divergence from the Caucasian wild goats might be considered distinct species.

Collectively, our results suggest that living representatives of the new lineage reported by Daly et al. (2022) might still exist and that they are not closely related to the Caucasian species, contrary to the findings of Daly et al. (2022). Furthermore, the genetic distances within the Caucasian lineage are very low (K‐2P = 0.25%), indicating a high degree of genetic similarity among the samples from the Konya, Antalya, and Direkli Cave populations. Additionally, the genetic distance between the samples forming this lineage and the two wild goat species from the Caucasus ( *C. caucasica* and *C. cylindricornis* ) was lower than the genetic distance between the lineage containing the new lineage of Daly et al. (2022) and Artvin sample and the two wild goat species from the Caucasus, with distances ranging from approximately 1.37% to 1.45%. The close phylogenetic relationship observed within both the Aegagrus and Caucasian lineages (living and ancient wild goats from Türkiye) could be associated with the Boreal‐Alpine distribution of wild goats. In this context, the mountain ranges known as the “Anatolian Diagonal”, which extend one after the other in a line from the southwest to the northeast of Türkiye, are thought to be geographical elements that have served as a dispersal‐allowing corridor for these populations living on steep rocky elevations (Ansell et al. 2011; Kornilios et al. 2011; Ahmadzadeh et al. 2013; Kapli et al. 2013; Şeker et al. 2018). By evaluating the findings of the previous paleogenomic study (Daly et al. 2022) alongside the current one, it can be inferred that there may be two distinct subpopulations of wild goats living in Türkiye, one related to *C. aegagrus* and the other to wild goats in the Caucasus, as previously suggested (Balkız 2002; Daly et al. 2022). The topologies of the BI and ML phylogenetic trees (Figure 2 and Supporting Information File 2) and the K‐2P genetic distances (Tables 5 and 6) indicate a possible difference at the subspecies level (K‐2P < 3%) according to the “genetic species concept” (Bradley and Baker 2001).

The date of evolutionary diversification of the Caprinae subfamily has been studied using molecular methods, such as allozyme variations and DNA sequencing (Randi et al. 1991; Lalueza‐Fox et al. 2005; Ropiquet and Hassanin 2005a, 2005b; Hassanin et al. 2012). These studies suggest that the adaptive radiation of Caprinae began in the Late Miocene on the Asian continent, with the common ancestor spreading across Eurasia and into Africa (Ropiquet and Hassanin 2005a). The ability to climb, which evolved in connection with environmental changes following the Late Miocene adaptive radiation, is believed to have played a key role in accelerating the diversification of the Caprinae. This process was further influenced by faunal migrations into suitable environments after the decrease in water levels during the Tortonian (7.8–7.6 Mya) and Messinian (6–5.3 Mya) salinity crises (Krijgsman et al. 1999, 2000). Estimates for the timing of evolutionary divergence within Caprinae vary across studies. Randi et al. (1991) suggested that divergence occurred around 5.28–7.08 Mya, while other studies proposed divergence began around 6.2 Mya (Lalueza‐Fox et al. 2005) or 10.96 Mya (Ropiquet and Hassanin 2005a). More recent analyses using complete mitochondrial genome data have estimated the divergence to have occurred approximately 9.2 Mya (Hassanin et al. 2012). The BI estimates in the current study revealed the evolutionary diversification of Caprinae began at around 8.18 Mya (95% HPD: 4.33–11.99 Mya, BPP: 1). This timing aligned well with previous molecular studies, confirming that diversification began in the Late Miocene. Guided by these results, we determined that the first diversification within the genus *Capra* began approximately 3.22 Mya (95% HPD: 1.69–4.72 Mya, BPP: 1), marked by the divergence of *C. sibirica* and *H. jemlahicus* from other species. This result closely aligned with the evolutionary divergence time (app. 3 ± 0.5 Mya, HPD: 2–4.1 Mya) determined by Ropiquet and Hassanin (2005a) for the differentiation of *C. sibirica* and *H. jemlahicus* from other species. This time period coincides with the beginning of climatic fluctuations and glacial cycles in the Early Pliocene and into the Early Pleistocene. During these periods, adverse conditions, including cyclical climate fluctuations characterized by repeated global cooling and increased glaciations, led to a decline in global temperatures and water scarcity. These factors significantly impacted the distribution of species and accelerated their evolutionary divergence (Hewitt 2004). Climatic changes, including glacial and interglacial cycles, that started prior to the Pliocene/Pleistocene transition are thought to have significantly influenced the evolutionary diversification within *Capra* (Ropiquet and Hassanin 2005a). Also, molecular evidence has suggested that new mitochondrial DNA, transferred through hybridization from the common ancestor of wild goats during the Plio‐Pleistocene, enhanced adaptation to life at high altitudes, providing a selective advantage to wild goat populations and triggering their subsequent adaptive radiation (Ropiquet and Hassanin 2005a). The diversification of the Caucasian lineage, comprising *C. aegagrus* , *C. cylindricornis* , and *C. caucasica* , occurred approximately 1.16 Mya with a 95% HPD: 0.60–1.69 Mya and BPP: 1. This time frame corresponded to the Calabrian stage of the Pleistocene (between 1.8 Mya and 774 ± 5 Kya). Similarly, the diversification of the Aegagrus lineage, which gave rise to domestic goats, also aligned with the Calabrian stage of the Pleistocene, occurring around 0.89 Mya (95% HPD: 0.47–1.31 Mya, BPP: 1). These estimates of diversification times aligned with the results of previous studies, which suggested that the rapid evolutionary diversification of wild goats likely occurred during the Plio‐Pleistocene (Ropiquet and Hassanin 2005a, 2006).

Although *C. hircus* was domesticated approximately 10,000–11,000 years ago, mitochondrial DNA studies suggested that its evolutionary divergence may have occurred much earlier, between 840,000 and 103,000 years ago (Luikart et al. 2001; Sultana et al. 2003; Joshi et al. 2004; Chen et al. 2005; Nomura et al. 2013; Colli et al. 2015). The results of this study indicated that *C. aegagrus* and *C. hircus* began diverging approximately 0.89 Mya, while genetic diversification within *C. hircus* started around 0.29 Mya (95% HPD: 0.15–0.42 Mya, BPP: 1). These estimates are considerably older than the onset of goat domestication in Anatolia during the Neolithic period, and their confidence intervals overlapped with the findings of previous studies. Past studies have dated the divergence of the domestic goat to approximately the Late Pleistocene (126 Kya–11.7 Kya), well before the onset of goat domestication (Luikart et al. 2001; Sultana et al. 2003; Joshi et al. 2004; Chen et al. 2005; Nomura et al. 2013; Colli et al. 2015). It is widely accepted that the mutation rate in mitochondrial DNA is approximately 2% per million years (Johns and Avise 1998; Taberlet et al. 1998; but see Nabholz et al. 2008). Given this general consensus, the evolutionary divergence times estimated in this study aligned closely with the genetic distances calculated using the Kimura 2‐parameter model. Another factor supporting this finding was the high BPP values computed for the main nodes in the BI phylogenetic tree. In this study, one specimen of *R. rupicapra* was placed within the *R. rupicapra* lineage, as anticipated. Consistent with the estimates of Lalueza‐Fox et al. (2005), the divergence between *R. rupicapra* and *R. pyrenaica* was estimated to have occurred approximately 1.5 Mya (95% HPD: 0.77–2.19 Mya and BPP: 1). Masini and Lovari (1988) stated that diversification of the *Rupicapra* lineage occurred during the Middle and Late Pleistocene in west Eurasia. Our results contradicted those of Masini and Lovari (1988), suggesting that the divergence between the two *Rupicapra* species may have occurred much earlier than previously believed, during the Early–Middle Pleistocene transition, as also proposed by Lalueza‐Fox et al. (2005).

## 5 Conclusions

The current study provided valuable new insights into the genetic diversity, phylogeny, and evolutionary history of domestic goats from Türkiye and Iraq, as well as wild goat ( *Capra aegagrus* ) and chamois ( *Rupicapra rupicapra* ) from Türkiye. The high genetic diversity of domestic goats in Türkiye supports its role as a potential center of early Neolithic domestication, though limited sampling from nearby regions calls for caution and further comparative research. Phylogenetic and phylogeographic analyses revealed a complex genetic structure between the Turkish and the Iraqi domestic goat breeds, with a low level of differentiation likely due to extensive gene flow and the widespread distribution of haplogroups. The present study also proposed that domestic goats in Türkiye might have originated from wild goats in Eastern Anatolia, supporting previous findings on the geographical origin of domestic goats. Molecular clock estimates of evolutionary divergence provided a deeper understanding of the timeline of goat evolution, showing that divergence within the genus *Capra* occurred well before the domestication process. The evolutionary separation times suggest that the Pliocene cooling and Pleistocene glacial cycles had a significant impact on the evolutionary differentiation of the *Capra* genus. The Pliocene cooling, beginning around 3 million years ago, likely accelerated the evolutionary divergence of goats by exposing them to various environmental pressures, such as habitat reductions and significant habitat changes (Sosdian and Rosenthal 2009; Ropiquet and Hassanin 2005a). The climatic cycles during the Pleistocene may have caused population isolation, the formation of new ecological niches, and the development of new morphological traits, leading to speciation and adaptation in wild goats (Hewitt 2004; Ropiquet and Hassanin 2005a). The diversification of the Caucasian lineage ( *C. aegagrus* , *C. cylindricornis* , and *C. caucasica* ) around 1.16 Mya aligns with the Calabrian phase of the Pleistocene, a period of substantial climatic shifts. Similarly, the Aegagrus lineage, which gave rise to domestic goats, likely underwent diversification during the same Pleistocene period, around 0.89 Mya, driven by these cyclical climate changes. In light of these findings, it can be concluded that the Pliocene cooling and Pleistocene glacial cycles played crucial roles in the evolutionary differentiation of *Capra* species, influencing the development of distinct lineages. These climatic changes, as indicated by molecular dating analyses, were important processes that facilitated the adaptation of populations to new environments, isolated populations, and promoted speciation, ultimately contributing to the emergence of both wild and domestic goats. Overall, our findings contributed to a more comprehensive understanding of goat domestication, genetic diversity, and evolutionary events, offering valuable data for future studies on the radiation and genetic adaptation of domestic goats worldwide. Detailed analyses with comprehensive sampling and the inclusion of additional molecular markers are necessary to fully clarify the taxonomic status of the two distinct wild goat populations in Türkiye—one related to *C. aegagrus* and the other to the wild goats of the Caucasian region. Further research will help to resolve their evolutionary relationships and provide a clearer understanding of their genetic differentiation.

## Author Contributions

**Saffet Teber:** conceptualization (equal), data curation (equal), formal analysis (equal), funding acquisition (lead), methodology (equal), software (equal), visualization (equal), writing – original draft (equal), writing – review and editing (equal). **Husham Abdulrahman Mahdi Al‐Abbasi:** conceptualization (equal), investigation (equal), validation (equal), writing – review and editing (equal). **Perinçek Seçkinozan Şeker:** conceptualization (equal), formal analysis (equal), funding acquisition (equal), investigation (equal), resources (equal), supervision (equal), validation (equal), writing – original draft (equal), writing – review and editing (equal). **Klaus‐Peter Koepfli:** conceptualization (equal), supervision (equal), validation (equal), writing – original draft (equal), writing – review and editing (equal). **Ahmet Yesari Selçuk:** conceptualization (equal), formal analysis (equal), investigation (equal), resources (equal), writing – review and editing (equal). **Mehmet Baran:** data curation (equal), formal analysis (equal), investigation (equal), methodology (equal), software (equal), validation (equal), visualization (equal), writing – review and editing (equal). **Coşkun Tez:** conceptualization (equal), formal analysis (equal), funding acquisition (equal), investigation (equal), resources (equal), supervision (equal), validation (equal), writing – original draft (equal), writing – review and editing (equal). **Osman Ibiş:** conceptualization (equal), data curation (lead), formal analysis (lead), funding acquisition (lead), investigation (equal), methodology (equal), project administration (lead), resources (equal), software (equal), supervision (equal), validation (equal), visualization (equal), writing – original draft (equal), writing – review and editing (equal).

## Ethics Statement

Research ethics committee approval was provided for the capture of the animals, permission No. E‐21264211‐288.04‐10822834 from the Republic of Turkey Ministry of Agriculture and Forestry, General Directorate of Nature Conservation and National Parks in regard to 23/061 numbered in conjunction with Erciyes University Local Ethics Committee for Animal Experiments.

## Conflicts of Interest

The authors declare no conflicts of interest.

## Supporting information


**Supplementary file 1.** Information on Mitogenome sequences of *Capra* species presented by this study and the representative species of the genera belongs to the subfamily Caprinae retrieved from GenBank. Geographic locations of the samples and GenBank accession numbers are listed.

**Supplementary file 2.** ML phylogenetic tree reconstructed using mitogenomes among the Caprinae lineages.

## Data Availability

The Turkish and Iraqi goat mitogenome sequencing data are available at NCBI‐GenBank: PV138188‐PV138217.
