# Supplementary material for: Phylogenetic Relationships and Evolutionary History of Goats (Mammalia: Capra) From Türkiye and Iraq, Inferred From Complete Mitochondrial Genomes
Source: Ecol Evol. 2025 Aug 13;15(8):e71985. doi: 10.1002/ece3.71985 (PMC12349903; doi:10.1002/ece3.71985)
Supplement: Supplementary file 1 — Information on Mitogenome sequences of Capra species presented by this study and the representative species of the genera belongs to the subfamily Caprinae retrieved from GenBank. Geographic locations of the samples and GenBank accession numbers are listed. [file ECE3-15-e71985-s002.docx]

**Supplement file 1.** Information on Mitogenome sequences of *Capra* species presented by this study and the representative species of the genera belongs to the subfamily Caprinae retrieved from GenBank. Geographic locations of the samples and GenBank accession numbers are listed.

| **Accession no.** | **Taxon** | **Breed** | **Haplogroup** | **Locality** | **Reference** |
| --- | --- | --- | --- | --- | --- |
| ***Capra hircus*** | | | | | |
| **PV138189**  **(CaHiIRQ1)** | ***Capra hircus*** | **Local** |  | **Iraq, Samarra** | **This study** |
| **PV138190**  **(CaHiIRQ2)** | ***Capra hircus*** | **Unknown** |  | **Iraq, Hillah** | **This study** |
| **PV138211**  **(CaHiIRQ3)** | ***Capra hircus*** | **Local** |  | **Iraq, Hit** | **This study** |
| **(PV138191)**  **CaHiIRQ4** | ***Capra hircus*** | **Cyprus** |  | **Iraq, Hit** | **This study** |
| **PV138193**  **(CaHiIRQ5)** | ***Capra hircus*** | **Shami** |  | **Iraq, Hit** | **This study** |
| **PV138194**  **(CaHiIRQ6)** | ***Capra hircus*** | **Cyprus** |  | **Iraq, Hit** | **This study** |
| **PV138210**  **(CaHiIRQ7)** | ***Capra hircus*** | **Shami** |  | **Iraq, Hit** | **This study** |
| **PV138209**  **(CaHiIRQ8)** | ***Capra hircus*** | **Local** |  | **Iraq, Jamiah** | **This study** |
| **PV138192**  **(CaHiIRQ9)** | ***Capra hircus*** | **Local** |  | **Iraq, Jamiah** | **This study** |
| **PV138202**  **(CaHiIRQ10)** | ***Capra hircus*** | **Local** |  | **Iraq, Hit** | **This study** |
| **PV138200**  **(CaHiIRQ11)** | ***Capra hircus*** | **Unknown** |  | **Iraq, Hillah** | **This study** |
| **PV138199**  **(CaHiIRQ12)** | ***Capra hircus*** | **Local** |  | **Iraq, Hillah** | **This study** |
| **PV138208**  **(CaHiIRQ13)** | ***Capra hircus*** | **Cyprus** |  | **Iraq, Hadi** | **This study** |
| **PV138201**  **(CaHiIRQ14)** | ***Capra hircus*** | **Unknown** |  | **Iraq, Hillah** | **This study** |
| **PV138204**  **(CaHiIRQ15)** | ***Capra hircus*** | **Unknown** |  | **Iraq, Hillah** | **This study** |
| **PV138207**  **(CaHiIRQ16)** | ***Capra hircus*** | **Unknown** |  | **Iraq, Hillah** | **This study** |
| **PV138212**  **(CaHiIRQ17)** | ***Capra hircus*** | **Unknown** |  | **Iraq, Hillah** | **This study** |
| **PV138203**  **(CaHiIRQ18)** | ***Capra hircus*** | **Unknown** |  | **Iraq, Hillah** | **This study** |
| **PV138197**  **(CaHiTR1)** | ***Capra hircus*** | **Turkish Saanen** |  | **Türkiye, Kirklareli** | **This study** |
| **PV138198**  **(CaHiTR2)** | ***Capra hircus*** | **Hair** |  | **Türkiye, Adana** | **This study** |
| **PV138195**  **(CaHiTR3)** | ***Capra hircus*** | **Hair** |  | **Türkiye, Adana** | **This study** |
| **PV138196**  **(CaHiTR4)** | ***Capra hircus*** | **Hair** |  | **Türkiye, Adana** | **This study** |
| **PV138206**  **(CaHiTR5)** | ***Capra hircus*** | **Turkish Saanen** |  | **Türkiye, Yozgat** | **This study** |
| **(PV138205)**  **CaHiTR6** | ***Capra hircus*** | **Angora** |  | **Türkiye, Ankara** | **This study** |
| **PV138214**  **(CaHiTR7)** | ***Capra hircus*** | **Hair** |  | **Türkiye, Kayseri** | **This study** |
| **PV138213**  **(CaHiTR8)** | ***Capra hircus*** | **Kilis** |  | **Türkiye, Kayseri** | **This study** |
| KR059151 | *Capra hircus* | Abaza | A1a | Türkiye | Colli et al., (2015) |
| KR059178 | *Capra hircus* | Abaza | A6 | Türkiye | Colli et al., (2015) |
| KR059186 | *Capra hircus* | Abaza | A | Türkiye | Colli et al., (2015) |
| KR059200 | *Capra hircus* | Angora | A | Türkiye | Colli et al., (2015) |
| KR059201 | *Capra hircus* | Angora | A | Türkiye | Colli et al., (2015) |
| KR059214 | *Capra hircus* | Angora | G | Türkiye | Colli et al., (2015) |
| KR059153 | *Capra hircus* | Baladie | A2 | Jordon | Colli et al., (2015) |
| KR059160 | *Capra hircus* | Baladie | A2a1 | Jordon | Colli et al., (2015) |
| KR059175 | *Capra hircus* | Baladie | A5 | Jordon | Colli et al., (2015) |
| KR059183 | *Capra hircus* | Baladie | A | Jordon | Colli et al., (2015) |
| KR059184 | *Capra hircus* | Beshi | A | Saudi Arabia | Colli et al., (2015) |
| KR059176 | *Capra hircus* | Girgentana | A5 | Italy | Colli et al., (2015) |
| KR059150 | *Capra hircus* | Grisons Striped | A1a | Switzerland | Colli et al., (2015) |
| KR059161 | *Capra hircus* | Gurku | A2a1 | Türkiye | Colli et al., (2015) |
| KR059203 | *Capra hircus* | Gurku | A | Türkiye | Colli et al., (2015) |
| KR059152 | *Capra hircus* | Hair | A2 | Türkiye | Colli et al., (2015) |
| KR059158 | *Capra hircus* | Hair | A2a1 | Türkiye | Colli et al., (2015) |
| KR059204 | *Capra hircus* | Hair | A | Türkiye | Colli et al., (2015) |
| KR059215 | *Capra hircus* | Hair | G | Türkiye | Colli et al., (2015) |
| KR059208 | *Capra hircus* | Kermanshah | A | Iran | Colli et al., (2015) |
| KR059213 | *Capra hircus* | Kermanshah | G | Iran | Colli et al., (2015) |
| KR059218 | *Capra hircus* | Khalkhali | G | Iran | Colli et al., (2015) |
| KR059191 | *Capra hircus* | Kurdi | A | Iran | Colli et al., (2015) |
| KR059211 | *Capra hircus* |  | D1 | Kyrgyzstan | Colli et al., (2015) |
| KR059212 | *Capra hircus* |  | D1 | Kyrgyzstan | Colli et al., (2015) |
| KR059220 | *Capra hircus* |  | B1 | Malaysia | Colli et al., (2015) |
| KR059146 | *Capra hircus* | Mati | A1 | Albania | Colli et al., (2015) |
| KR059192 | *Capra hircus* | Naini | A | Iran | Colli et al., (2015) |
| KR059154 | *Capra hircus* | Najrani | A2 | Saudi Arabia | Colli et al., (2015) |
| KR059225 | *Capra hircus* | Payoya | C1a | Spain | Colli et al., (2015) |
| KR059172 | *Capra hircus* | Peacock | A4 | Switzerland | Colli et al., (2015) |
| KR059180 | *Capra hircus* | Pinzgau | A7 | Austria | Colli et al., (2015) |
| KR059167 | *Capra hircus* | Swiss Alpine | A3 | Switzerland | Colli et al., (2015) |
| KR059171 | *Capra hircus* | Swiss Alpine | A4 | Switzerland | Colli et al., (2015) |
| KR059223 | *Capra hircus* | Swiss Alpine | C1a | Switzerland | Colli et al., (2015) |
| KR059224 | *Capra hircus* | Swiss Alpine | C1a | Switzerland | Colli et al., (2015) |
| KR059206 | *Capra hircus* | Taleshi | A | Iran | Colli et al., (2015) |
| KR059216 | *Capra hircus* |  | G | Iran | Colli et al., (2015) |
| KR059157 | *Capra hircus* | Zaraibi | A2a1 | Egypt | Colli et al., (2015) |
| KR059159 | *Capra hircus* | Zaraibi | A2a1 | Egypt | Colli et al., (2015) |
| KR059155 | *Capra hircus* | Zaraibi | A2a | Egypt | Colli et al., (2015) |
| KR059188 | *Capra hircus* | Zaraibi | A | Egypt | Colli et al., (2015) |
| ***Capra aegagrus, C. caucasica, C. cylindricornis, C. falconeri, C. ibex, C. nubiana, C. pyrenaica, C. sibirica*** | | | | | |
| **PV138188**  **(CaAeTR1)** | ***Capra aegagrus***  **(Aegagrus lineage)** |  |  | **Türkiye, Artvin** | **This study** |
| KT290893 | *Capra aegagrus* |  |  |  | Zhang et al. (2015), Unpublished |
| LR884221 | *Capra aegagrus* |  |  |  | Daly (2020), Unpublished |
| LS992603 | *Capra aegagrus* |  |  |  | Daly (2018), Unpublished |
| LS992607 | *Capra aegagrus* |  |  |  | Daly (2018), Unpublished |
| LS992617 | *Capra aegagrus* |  |  |  | Daly (2018), Unpublished |
| OW568852 | *Capra aegagrus* |  |  |  | Daly (2022), Unpublished |
| OW568859 | *Capra aegagrus* |  |  |  | Daly (2022), Unpublished |
| OW568907 | *Capra aegagrus* |  |  |  | Daly (2022), Unpublished |
| OW568911 | *Capra aegagrus* |  |  |  | Daly (2022), Unpublished |
| OW568916 | *Capra aegagrus* |  |  |  | Daly (2022), Unpublished |
| KR059219 | *Capra aegagrus* | Bezoar | B | Iran | Colli et al., (2015) |
| KR059222 | *Capra aegagrus* | Bezoar | C1 | Iran | Colli et al., (2015) |
| KR059221 | *Capra aegagrus* | Bezoar | C | Iran | Colli et al., (2015) |
| KR059210 | *Capra aegagrus* | Bezoar | D | Iran | Colli et al., (2015) |
| KR059226 | *Capra aegagrus* | Bezoar | F | Iran | Colli et al., (2015) |
| **PV138215**  **(CaCaTR1)** | ***Capra aegagrus***  **(Caucasian lineage)** |  |  | **Türkiye, Antalya** | **This study** |
| **PV138216**  **(CaCaTR2)** | ***Capra aegagrus***  **(Caucasian lineage)** |  |  | **Türkiye, Konya** | **This study** |
| JN632609 | *Capra caucasica* |  |  |  | Hassanin et al., (2012) |
| OW568848 | *Capra cylindricornis* |  |  |  | Daly (2022), Unpublished |
| OW568849 | *Capra cylindricornis* |  |  |  | Daly (2022), Unpublished |
| OW568906 | *Capra cylindricornis* |  |  |  | Daly (2022), Unpublished |
| OW568856 | *Capra falconeri* |  |  |  | Daly (2022), Unpublished |
| FJ207525 | *Capra falconeri* |  |  |  | Hassanin et al., (2009) |
| OW568909 | *Capra ibex* |  |  |  | Daly (2022), Unpublished |
| OW568912 | *Capra ibex* |  |  |  | Daly (2022), Unpublished |
| FJ207526 | *Capra ibex* |  |  |  | Hassanin et al., (2009) |
| OW568908 | *Capra nubiana* |  |  |  | Daly (2022), Unpublished |
| FJ207527 | *Capra nubiana* |  |  |  | Hassanin et al., (2009) |
| OW568860 | *Capra pyrenaica* |  |  |  | Daly (2022), Unpublished |
| FJ207528 | *Capra pyrenaica* |  |  |  | Hassanin et al., (2009) |
| OW568913 | *Capra sibirica* |  |  |  | Daly (2022), Unpublished |
| FJ207529 | *Capra sibirica* |  |  |  | Hassanin et al., (2009) |
| ***Capricornis* sp.** | | | | | |
| NC_012096 | *Capricornis crispus* |  |  |  | Yasue et al., (2023) Unpublished |
| NC_023457 | *Capricornis milneedwardsii* |  |  |  | Gong et al., (2016) |
| NC_045205 | *Capricornis rubidus* |  |  |  | Mori et al., (2019) |
| NC_020629 | *Capricornis sumatraensis* |  |  |  | Hassanin et al., (2009) |
| NC_010640 | *Capricornis swinhoei* |  |  |  | Lee et al., (2023) Unpublished |
| ***Rupicapra rupicapra, R. pyrenaica*** | | | | | |
| **PV138217**  **(RuRuTR1)** | ***Rupicapra rupicapra*** |  |  | **Türkiye, Artvin** | **This study** |
| FJ207539 | *Rupicapra rupicapra* |  |  |  | Hassanin et al., (2009) |
| MW713530 | *Rupicapra rupicapra asiatica* |  |  | Türkiye | Pérez et al., (2022) |
| MW588899 | *Rupicapra rupicapra balcanica* |  |  |  | Iacolina et al., (2021) |
| MW713529 | *Rupicapra rupicapra balcanica* |  |  | North Macedonia | Pérez et al., (2022) |
| MW713528 | *Rupicapra rupicapra balcanica* |  |  | Serbia | Pérez et al., (2022) |
| KJ184175 | *Rupicapra rupicapra cartusiana* |  |  |  | Pérez et al., (2014) |
| MW713531 | *Rupicapra rupicapra caucasica* |  |  | Georgia | Pérez et al., (2022) |
| MW588900 | *Rupicapra rupicapra rupicapra* |  |  |  | Iacolina et al., (2021) |
| MW588901 | *Rupicapra rupicapra tatrica* |  |  |  | Iacolina et al., (2021) |
| MW713527 | *Rupicapra rupicapra tatrica* |  |  | Poland | Pérez et al., (2022) |
| FJ207538 | *Rupicapra pyrenaica* |  |  |  | Hassanin et al., (2009) |
| KJ184173 | *Rupicapra pyrenaica ornata* |  |  |  | Pérez et al., (2014) |
| MW713525 | *Rupicapra pyrenaica* |  |  | Spain | Pérez et al., (2022) |
| KJ184174 | *Rupicapra pyrenaica pyrenaica* |  |  |  | Pérez et al., (2014) |
| MW588895 | *Rupicapra pyrenaica pyrenaica* |  |  |  | Iacolina et al., (2021) |
| NC_016689 | *Pseudois schaeferi* |  |  |  | Zou et al., (2016) Unpublished |
| NC_020632 | *Pseudois nayaur* |  |  |  | Hassanin et al., (2009) |
| ***Ovis, Ovibos, Oreamnos, Naemorhedus, Myotragus, Hemitragus, Ammotragus, Arabitragus, Bootherium, Budorcas*** | | | | | |
| NC_026064 | *Ovis vignei* | Urial |  |  | Lv et al., (2015) |
| NC_026063 | *Ovis orientalis* | Asian mouflon |  |  | Lv et al., (2015) |
| NC_039431 | *Ovis nivicola lydekkeri* | Yakut snow sheep |  | Russia | Dotsev et al., (2019) |
| NC_039432 | *Ovis dalli* |  |  | USA | Dotsev et al., (2019) |
| NC_015889 | *Ovis canadensis* |  |  |  | Miller et al., (2012) |
| NC_020656 | *Ovis ammon* |  |  |  | Meadows et al., (2011) |
| NC_001941 | *Ovis aries* |  |  |  | Hiendleder et al., (1998) |
| NC_020631 | *Ovibos moschatus* |  |  |  | Hassanin et al., (2009) |
| KF826487 | *Ovibos moschatus* |  |  |  | Lipinski et al., (2014) Unpublished |
| NC_020630 | *Oreamnos americanus* |  |  |  | Hassanin et al., (2009) |
| NC_020723 | *Naemorhedus griseus* |  |  |  | Hassanin et al., (2012) |
| NC_021381 | *Naemorhedus goral* |  |  |  | Yang et al., (2013) |
| MN853098 | *Naemorhedus cranbrooki* |  |  | Myanmar | Li et al., (2020) |
| NC_013751 | *Naemorhedus caudatus* |  |  |  | Jang & Hwang (2010) |
| NC_020722 | *Naemorhedus baileyi* |  |  |  | Hassanin et al., (2012) |
| NC_042943 | *Myotragus balearicus* |  |  | Spain | Bover et al., (2019) |
| MK847862 | *Myotragus balearicus* |  |  | Spain | Bover et al., (2019) |
| NC_020628 | *Hemitragus jemlahicus* |  |  |  | Hassanin et al., (2009) |
| NC_020621 | *Hemitragus jayakari* |  |  |  | Hassanin et al., (2009) |
| FJ207522 | *Ammotragus lervia* |  |  |  | Hassanin et al., (2009) |
| NC_009510 | *Ammotragus lervia* |  |  |  | Mereu et al., (2008) |
| MN971587 | *Arabitragus jayakari* |  |  |  | Al-Rawahi et al., (2022) |
| MH706736 | *Bootherium bombifrons* |  |  | Canada | Bover et al., (2018) |
| NC_044933 | *Bootherium bombifrons* |  |  | USA | Bover et al., (2018) |
| NC_039686 | *Budorcas taxicolor tibetana* |  |  | China | Zhou et al., (2019) |
| NC_043930 | *Budorcas taxicolor taxicolor* |  |  | India | Kumar et al., (2019) |
| **OutGroup-*Bos*** | | | | | |
| NC_013996 | *Bos primigenius* |  |  |  | Edwards et al., (2010) |
| NC_006853 | *Bos taurus* |  |  |  | Chung, (2013) |

**References**

Colli, L., Lancioni, H., Cardinali, I., Olivieri, A., Capodiferro, M. R., Pellecchia, M., ... & Achilli, A. (2015). Whole mitochondrial genomes unveil the impact of domestication on goat matrilineal variability. *BMC genomics*, *16*, 1-12. <https://doi.org/10.1186/s12864-015-2342-2>

Hassanin, A., Delsuc, F., Ropiquet, A., Hammer, C., Van Vuuren, B. J., Matthee, C., ... & Couloux, A. (2012). Pattern and timing of diversification of Cetartiodactyla (Mammalia, Laurasiatheria), as revealed by a comprehensive analysis of mitochondrial genomes. *Comptes rendus biologies*, *335*(1), 32-50. <https://doi.org/10.1016/j.crvi.2011.11.002>

Hassanin, A., Ropiquet, A., Couloux, A., & Cruaud, C. (2009). Evolution of the mitochondrial genome in mammals living at high altitude: new insights from a study of the tribe Caprini (Bovidae, Antilopinae). *Journal of molecular evolution*, *68*, 293-310. <https://doi.org/10.1007/s00239-009-9208-7>

Pérez, T., Fernández, M., Palacios, B., & Domínguez, A. (2022). Phylogenetic analysis of the complete mitochondrial genomes in the ten Rupicapra subspecies and implications for the existence of multiple glacial refugia in Europe. *Animals*, *12*(11), 1430. <https://doi.org/10.3390/ani12111430>

Iacolina, L., Buzan, E., Safner, T., Bašić, N., Geric, U., Tesija, T., ... & Šprem, N. (2021). A mother’s story, mitogenome relationships in the genus Rupicapra. *Animals*, *11*(4), 1065. <https://doi.org/10.3390/ani11041065>

Pérez, T., González, I., Essler, S. E., Fernández, M., & Domínguez, A. (2014). The shared mitochondrial genome of Rupicapra pyrenaica ornata and Rupicapra rupicapra cartusiana: Old remains of a common past. *Molecular phylogenetics and evolution*, *79*, 375-379. <https://doi.org/10.1016/j.ympev.2014.07.004>

Lv, F. H., Peng, W. F., Yang, J., Zhao, Y. X., Li, W. R., Liu, M. J., ... & Li, M. H. (2015). Mitogenomic meta-analysis identifies two phases of migration in the history of eastern Eurasian sheep. *Molecular biology and evolution*, *32*(10), 2515-2533. <https://doi.org/10.1093/molbev/msv139>

Dotsev, A. V., Kunz, E., Shakhin, A. V., Petrov, S. N., Kostyunina, O. V., Okhlopkov, I. M., ... & Zinovieva, N. A. (2019). The first complete mitochondrial genomes of snow sheep (Ovis nivicola) and thinhorn sheep (Ovis dalli) and their phylogenetic implications for the genus Ovis. *Mitochondrial DNA Part B*, *4*(1), 1332-1333. <https://doi.org/10.1080/23802359.2018.1535849>

Miller, J. M., Malenfant, R. M., Moore, S. S., & Coltman, D. W. (2012). Short reads, circular genome: skimming SOLiD sequence to construct the bighorn sheep mitochondrial genome. *Journal of Heredity*, *103*(1), 140-146. <https://doi.org/10.1093/jhered/esr104>

Meadows, J. R. S., Hiendleder, S., & Kijas, J. W. (2011). Haplogroup relationships between domestic and wild sheep resolved using a mitogenome panel. *Heredity*, *106*(4), 700-706. <https://doi.org/10.1038/hdy.2010.122>

Hiendleder, S., Lewalski, H., Wassmuth, R., & Janke, A. (1998). The complete mitochondrial DNA sequence of the domestic sheep (Ovis aries) and comparison with the other major ovine haplotype. *Journal of Molecular Evolution*, *47*, 441-448. <https://doi.org/10.1007/PL00006401>

Hassanin, A., Delsuc, F., Ropiquet, A., Hammer, C., Van Vuuren, B. J., Matthee, C., ... & Couloux, A. (2012). Pattern and timing of diversification of Cetartiodactyla (Mammalia, Laurasiatheria), as revealed by a comprehensive analysis of mitochondrial genomes. *Comptes rendus biologies*, *335*(1), 32-50. <https://doi.org/10.1016/j.crvi.2011.11.002>

Yang, C., Xiang, C., Qi, W., Xia, S., Tu, F., Zhang, X., ... & Yue, B. (2013). Phylogenetic analyses and improved resolution of the family Bovidae based on complete mitochondrial genomes. *Biochemical Systematics and Ecology*, *48*, 136-143. <https://doi.org/10.1016/j.bse.2012.12.005>

Li, G., Sun, N., Swa, K., Zhang, M., Lwin, Y. H., & Quan, R. C. (2020). Phylogenetic reassessment of gorals with new evidence from northern Myanmar reveals five distinct species. *Mammal Review*, *50*(4), 325-330. <https://doi.org/10.1111/mam.12200>

Jang, K. H., & Hwang, U. W. (2010). Complete mitochondrial genome of the Korean goral Naemorhaedus caudatus (Ruminantia, Bovidae, Antilopinae) and conserved domains in the control region of Caprini. *Mitochondrial DNA*, *21*(3-4), 62-64. <https://doi.org/10.3109/19401736.2010.490833>

Bover, P., Llamas, B., Mitchell, K. J., Thomson, V. A., Alcover, J. A., Lalueza-Fox, C., ... & Pons, J. (2019). Unraveling the phylogenetic relationships of the extinct bovid Myotragus balearicus Bate 1909 from the Balearic Islands. *Quaternary Science Reviews*, *215*, 185-195. <https://doi.org/10.1016/j.quascirev.2019.05.005>

Gong, S., Peng, R., Jiang, L., Deng, S., & Zou, F. (2016). The complete mitochondrial genome sequence of the Chinese Serow, Capricornis milneedwardsii (Cetartiodactyla: Caprinae). *Mitochondrial DNA Part A*, *27*(1), 54-55. <https://doi.org/10.3109/19401736.2013.873896>

Mori, E., Nerva, L., & Lovari, S. (2019). Reclassification of the serows and gorals: the end of a neverending story?. *Mammal Review*, *49*(3), 256-262. <https://doi.org/10.1111/mam.12154>

Mereu, P., Palici di Suni, M., Manca, L., & Masala, B. (2008). Complete nucleotide mtDNA sequence of Barbary sheep (Ammotragus lervia) Full Length Research Paper. *DNA Sequence*, *19*(3), 241-245. <https://doi.org/10.1080/10425170701550599>

Bover, P., Llamas, B., Thomson, V. A., Pons, J., Cooper, A., & Mitchell, K. J. (2018). Molecular resolution to a morphological controversy: The case of North American fossil muskoxen Bootherium and Symbos. *Molecular phylogenetics and evolution*, *129*, 70-76. <https://doi.org/10.1016/j.ympev.2018.08.008>

Edwards, C. J., Magee, D. A., Park, S. D., McGettigan, P. A., Lohan, A. J., Murphy, A., ... & MacHugh, D. E. (2010). A complete mitochondrial genome sequence from a mesolithic wild aurochs (Bos primigenius). *PLoS One*, *5*(2), e9255. <https://doi.org/10.1371/journal.pone.0009255>

Zhou, M., Yu, J., Li, B., Ouyang, B., & Yang, J. (2019). The complete mitochondrial genome of Budorcas taxicolor tibetana (Artiodactyla: Bovidae) and comparison with other Caprinae species: Insight into the phylogeny of the genus Budorcas. *International journal of biological macromolecules*, *121*, 223-232. <https://doi.org/10.1016/j.ijbiomac.2018.10.020>

Kumar, A., Gautam, K. B., Singh, B., Yadav, P., Gopi, G. V., & Gupta, S. K. (2019). Sequencing and characterization of the complete mitochondrial genome of Mishmi takin (Budorcas taxicolor taxicolor) and comparison with the other Caprinae species. *International journal of biological macromolecules*, *137*, 87-94. <https://doi.org/10.1016/j.ijbiomac.2019.06.201>

Al-Rawahi, A. N., Alalawi, Z. S., Asaf, S., Khan, A. L., Khan, A., Al-Rawahi, H., ... & Al-Harrasi, A. (2022). Complete mitochondrial genome of endangered Arabian tahr (Arabitragus jayakari) and phylogenetic placement. *Mitochondrial DNA Part B*, *7*(6), 1189-1190. <https://doi.org/10.1080/23802359.2022.2090295>

Chung, H. (2013). Phylogenetic analysis and characterization of mitochondrial DNA for Korean native cattle. *Open Journal of Genetics*, *3*(1), 12-23. <http://dx.doi.org/10.4236/ojgen.2013.31003>
